# Supplementary material for: Colonization of intervertebral discs by Cutibacterium acnes in patients with low back pain: Protocol for an analytical study with microbiological, phenotypic, genotypic, and multiomic techniques
Source: PLoS One. 2023 Feb 27;18(2):e0271773. doi: 10.1371/journal.pone.0271773 (PMC9970075; doi:10.1371/journal.pone.0271773)
Supplement: S1 File — (PDF) [file pone.0271773.s002.pdf]

ClinicalTrials.gov Protocol Registration and Results System (PRS) Receipt
Release Date: October 11, 2021

ClinicalTrials.gov ID: NCT05090553

Study Identification

Unique Protocol ID: VMRDocProtocol_2021

Brief Title: Protocol for an Analytical Study With Microbiological, Phenotypic, Genotypic and Multiomics Techniques

Official Title: Cutibacterium Acnes Colonization on Intervertebral Discs of Patients With Low Back Pain: Protocol for an Analytical Study With Microbiological, Phenotypic, Genotypic and Multiomics Techniques

Secondary IDs:

Study Status

Record Verification: October 2021

Overall Status: Active, not recruiting

Study Start: August 1, 2021 [Actual]

Primary Completion: March 1, 2022 [Anticipated]

Study Completion: October 1, 2022 [Anticipated]

Sponsor/Collaborators

Sponsor: Gaffree & Guinle Universitary Hospital

Responsible Party: Principal Investigator
Investigator: Vinicius Magno da Rocha [vdarocha]
Official Title: Clinical Professor
Affiliation: Gaffree & Guinle Universitary Hospital

Collaborators: Coordenação de Aperfeiçoamento de Pessoal de Nível Superior.
Conselho Nacional de Desenvolvimento Científico e Tecnológico

Oversight

U.S. FDA-regulated Drug: No

U.S. FDA-regulated Device: No

U.S. FDA IND/IDE: No

Human Subjects Review: Board Status: Approved
Approval Number: 50077521.0.0000.5258
Board Name: Vinicius Magno da Rocha, Jorge Leite and Rossano Fiorelli
Board Affiliation: Research Ethics Committee of the Gaffrée & Guinle Universitary Hospital
Phone: 55 21 99700-7240

Email: professorviniciusmagno@edu.unirio.br
Address:

Rio de Janeiro, RJ, Brazil, 20270-004

Data Monitoring: No

FDA Regulated Intervention: No

Study Description

Brief Summary: The study aims to identify metabolites present in intervertebral discs colonized by C. acnes from patients with low back pain and degenerative disc disease, correlating them with their clinical, radiological and demographic profiles.

Detailed Description:

Conditions

Conditions: Back Pain, Low
Degenerative Disc Disease

Keywords:

Study Design

Study Type: Interventional

Primary Purpose: Basic Science

Study Phase: N/A

Interventional Study Model: Factorial Assignment

Number of Arms: 4

Masking: Quadruple (Participant, Care Provider, Investigator, Outcomes Assessor)
Neither the patient nor the surgeons will have access to the results of microbiological cultures or molecular analysis. The radiologist who will review the exams will also be blinded to patient data and laboratory results. The researcher who will analyze the pain and function scores will also be blinded.

Allocation: Randomized

Enrollment: 120 [Anticipated]

Arms and Interventions

Arms	Assigned Interventions
Active Comparator: surgical treatment<br>Lumbar Spinal Fusion Surgery	Procedure/Surgery: Lumbar Spinal Fusion Surgery<br>During surgery will be collected five disc fragments and five fragments of muscle-ligament tissue adjacent to the collected disc.
Experimental: Bacterial culture<br>Cutibacterium acnes culture in two different atmosphere:<br>• Carbon dioxide (CO_2) from 5% up to 10%<br>• CO_2 10%, H_2 10% and N_2 80%	Biological/Vaccine: Bacterial culture<br>Samples will be seeded on blood agar and anaerobic blood agar plates. After sowing in solid media, the swab will be inoculated in thioglycolate medium, where it will remain for 14 days
Experimental: Phenotypic and genotypic characterization	Genetic: Phenotypic and genotypic characterization

Arms	Assigned Interventions
Mass spectrometry and (Polimerase Chain Reaction) PCR	The phenotypic identification of the species will be carried out through mass spectrometry, with the MALDI-TOF equipment. PCR analyzes performed in two phases, the first to confirm the presence of bacteria (target and non-target) using primers capable of amplifying eubacteria ribosomal deoxyribonucleic acid (rDNA) 16s and the second directed to confirm C. acnes through specific rDNA 16s primers.
Experimental: Multiomics analysis<br>• Genome sequencing<br>• Proteomics<br>• Metabolomics	Genetic: Multiomics analysis<br>Whole Genome Sequencing, proteomics and metabolomics analysis

Outcome Measures

Primary Outcome Measure:

1. Incidence of C. acnes on intervertebral disc
   Incidence of C. acnes on intervertebral disc in patients with chronic low back pain and DDL.

   [Time Frame: Up to 5 months]

Eligibility

Minimum Age: 18 Years

Maximum Age: 65 Years

Sex: All

Gender Based: No

Accepts Healthy Volunteers: No

Criteria: Inclusion Criteria:

- low back pain lasting longer than 3 months;
- MRI findings of degenerative lumbar discopathy (DDL) performed less than 6 months after inclusion in the study;
- indication for open surgical treatment, with isolated microdiscectomy or associated with lumbar arthrodesis;
- failure of conservative treatment for at least 6 weeks and/or progressive neurological deficit

Exclusion Criteria:

- History of open lumbar spine surgery at any stage of life;
- presence of chemotherapy or pulse therapy with corticoids;
- patients with immune deficiency;
- patients undergoing previous intradiscal therapies (nucleotomy or discography);
- patients undergoing previous endoscopic surgery;
- patients with a history of previous spinal infection treated with antibiotics in the 6 months prior to inclusion in the study;
- use of antibiotics in the 2 months prior to the surgical procedure

Contacts/Locations

Central Contact Person: Vinicius M da Rocha, MD

Telephone: 55 21 99700-7240
Email: viniciusmagnodarocha@gmail.com

Central Contact Backup:

Study Officials:

Locations: **Brazil**
Hospital Universitário Gaffrée e Guinle
Rio de Janeiro, Brazil, 20270-004
Contact: Vinicius M da Rocha, MD 55 21 99700-7240
viniciusmagnodarocha@gmail.com

IPDSharing

Plan to Share IPD:

References

Citations:

Links:

Available IPD/Information:

U.S. National Library of Medicine | U.S. National Institutes of Health | U.S. Department of Health & Human Services
