# Supplementary material for: Colonization of intervertebral discs by Cutibacterium acnes in patients with low back pain: Protocol for an analytical study with microbiological, phenotypic, genotypic, and multiomic techniques
Source: PLoS One. 2023 Feb 27;18(2):e0271773. doi: 10.1371/journal.pone.0271773 (PMC9970075; doi:10.1371/journal.pone.0271773)
Supplement: S2 File — (PDF) [file pone.0271773.s003.pdf]

“COLONIZATION OF INTERVERTEBRAL DISCS BY *Cutibacterium acnes* IN PATIENTS WITH CHRONIC LUMBAR PAIN”

Vinícius Magno da Rocha
Carla Ormundo Gonçalves Ximenes Lima
André Xavier Bandeira
Eliane de Oliveira Ferreira
Mariana Balardino Bogado Faria
Rossano Kepler Alvim Fiorelli

Abstract

One of the most common reasons of chronic low back pain is lumbar disc degeneration. Its cause is thought to be complex, although its pathophysiological basis is unknown. Subclinical infection by *Cutibacterium acnes* has piqued the interest of disc degeneration researchers, who suspect that the presence of the microorganism in discs is linked to the production of inflammatory mediators associated with low back pain and/or implant failure in patients undergoing surgical treatment.

C. acnes identification from clinical specimens is a difficulty recognized to microbiologists and infectologists, requiring particular collection and culture techniques that are still not routinely used in most health care settings. Studies published before to the proposal of this study reveal disparate prevalence rates for C. acnes colonization of intervertebral discs, which we attribute to the variability of the methodologies used, or possibly to errors in clinical specimen collection and preparation. Thus, we propose an analytical cross-sectional study, in which clinical specimens of patients undergoing isolated lumbar discectomy surgeries will be analyzed, or in association with lumbar arthrodesis, between the months of August/2021 and August/2022.

Patients will be between the ages of 20 and 80, with MRI scans showing chronic low back pain and lumbar disc degeneration. Patients with a history of intervertebral disc infection and/or spondylodiscitis, as well as those who have already undergone discectomy procedures in the intervertebral disc collection area, will not be included in the study. Patients who have used oral or parenteral antibiotics in the fifteen days preceding the date of the surgical operation for which samples will be taken will also be excluded.

Skin swabs prior to surgical asepsis and antibiotic prophylaxis; 2 musculoskeletal tissue fragments surrounding the approaching disc; and 3 intervertebral disc fragments will be collected. Swab samples will be cultured on blood agar, anaerobic blood agar, and thioglycolate in a capnophilic environment. Vortexing will be used to prepare musculoligamentous tissue and intervertebral disc samples for inoculation. For a period of 14 days, all samples will be monitored. If no growth occurs, they will be reincubated and a new reading will be taken after 72 hours. The same method will be followed for all thioglycolate vials. Subcultures will be performed every 72 hours for the next three days, with the last subculture taking place on the 14th day of incubation in the same solid. We expect that by combining the aforementioned techniques, we would be able to acquire a larger prevalence of C. acnes colonization of the intervertebral discs as compared to results obtained using techniques traditionally employed for the identification of bacteria from musculoskeletal tissue specimens. Obtaining more consistent prevalence results may alter the course of

research into the pathophysiological bases of disc degeneration, directing research toward the set of alterations caused by the presence of the microorganism in the intervertebral disc, with the chronic process of low back pain and/or infectious complications post-operative events related to this bacterium.

Introduction

Low back pain is a common complaint in the population, resulting in a significant demand for health services as well as various losses associated with work leave, ranking second only to mental diseases in terms of impairing job productivity (1-4).

Degenerative disc disease is the most common cause of low back pain in adults (5, 6), and there is a large body of literature highlighting the relationship between inflammatory mediators and biomechanical stressors of the intervertebral disc as a driving force for the pathophysiological alterations observed in patients with disc degeneration and lumbar pain (7-11). Low-virulence anaerobic bacteria have recently gained prominence in the pathophysiology of lumbar degenerative disease (LDD), both because they cause a chronic inflammatory process in the intradiscal environment and because they favor cytoarchitecture failures of the intervertebral disc, which determines chronic pain and failure (12-16).

Many studies have verified the existence of anaerobic bacteria in intervertebral discs with DDL since the first isolation (17), with prevalence ranging from 13 to 44 percent (16, 18-21). Cutibacterium acnes is the most common species isolated among the most frequently isolated bacteria (19, 22).

C. acnes is a Gram-stainable, anaerobic, aerotolerant, pleomorphic, non-spore-forming bacillus-shaped bacterium. It is a species that lives in pilosebaceous follicles and the skin, and its population can outnumber that of coagulase negative Staphylococcus (23), accounting for half of the total microbiota in humans (24), particularly in areas with a high concentration of sebaceous glands (face, scalp, scalp, chest, and back) (25). In vitro isolation of C. acnes from tissue specimens with low oxygen tension, such as the intervertebral disc, is difficult, requiring care and specific handling and culture techniques (26).

C. acnes requires an environment and particular incubation media for anaerobic microorganisms, and the isolation period is longer due to its slow development than for bacteria usually isolated from musculoskeletal tissues (27). Other authors' diverse results in assessing the presence of this bacterium in musculoskeletal tissues may indicate faults in the sampling technique and specimen handling, as well as external contamination or even technical error during the complex laboratory treatment process. In the last decade, different authors have proposed changes in the collection and analysis protocols for orthopedic implants and specimens of musculoskeletal tissue for the isolation of *C. acnes*, such as (1) changes in the collection process of clinical specimens, (2) increased of the number of specimens collected, (3) extending the incubation period of the samples, (4) use of solid and liquid culture media in an anaerobic environment, (5) inoculation of macerated tissues, (6) sonication[1] and (7) use from the vortex[2]. (28-31)

Such changes are helping to change the epidemiological picture of C. acnes morbidity and mortality, as well as a better understanding of the pathophysiological bases in previously unknown scenarios, such as surgical implant failure and the occurrence of induced low back pain caused by the presence of this bacterium in the intervertebral disc (22, 32).The study proposed here aims to use a combination of

improvements in collection and analysis techniques aimed at isolating *C. acnes* from intervertebral discs of patients with LDD and low back pain.

[1] Conjunto formado por duas vértebras adjacentes e um disco intervertebral interposto. 12. Weiner SS, Nordin M. Prevention and management of chronic back pain. Best Pract Res Clin Rheumatol. 2010;24(2):267-79.

[2]Low-frequency ultrasound wave transmission technique, which promotes the release of energy and breaking the connection between the microorganism and the implant.

[3]Agitator composed of an engine that generates the movement of a receptacle made of synthetic rubber, where the vials or tubes with the substances that will be subjected to agitation are placed. A vibrating orbital movement is produced which creates a vortex in the liquid subjected to the apparatus.

Objectives

To determine the prevalence of *C. acnes* in samples of intervertebral discs from patients with lumbar degenerative disease and chronic low back pain who underwent surgical open discectomy.

Other objectives of the study are to outline a phylotypic profile of the most isolated C. acnes strains by bacterial WGS, and to identify a molecular profile for LDD and LBP in patients with positive cultures for C. acnes through proteomic and metabolomic techniques

Methodology

DESIGN

This is an analytical cross-sectional study, in which clinical specimens of patients undergoing isolated lumbar discectomy surgeries, or in association with lumbar arthrodesis, both by open approach, between the months of October/2021 and October/2022 will be analyzed.

SAMPLE SIZE CALCULATION

To calculate the sample size of the study proposed here, we started from the premise that, regardless of the presence of symptoms, the prevalence of DLD varies around 30% in the adult population. (2) To define the universe of interest, we also used the data that approximately 50% of the Brazilian population will have low back pain at some point in their lives. (1, 2, 4)

Thus, for the municipality of Rio de Janeiro, with an estimated population of 6,747,815 inhabitants according to the last population census, (33) of which 5,057,307 are over 20 years old, we infer that 1,517,192 have DLD and of these, 758,596 will have low back pain (N). For a margin of error (e) of 10%, standard deviation (P) of 0.5 and a confidence interval of 90%, we would have a z-score of 1.64 (Z). Applying these variables in the formula below, the sample size (n) would be 69 individuals.

$$n = \frac{Z^2 \times P\,(1-P)/\,e^2}{1 + \left[Z^2 \times \dfrac{P(1-P)}{e^2 \times N}\right]}$$

STUDY SITES

The collection of clinical specimens will be carried out at Hospital São Matheus (HSM), in a private partnership with the Intermedical Spine Surgery Group, without profit or conflicts of interest.

The analyzes of the clinical specimens will be carried out at the Anaerobic Biology Laboratory of the Paulo de Góes Institute of Microbiology located at the Health Sciences Center (CCS), at the Federal University of Rio de Janeiro (UFRJ).

CHARACTERIZATION OF PATIENTS

SELECTION CRITERIA

Patients with no gender restrictions, between 18 and 65 years of age, with degenerative lumbar disc disease and chronic low back pain, undergoing surgical treatment with isolated discectomy or associated with arthrodesis **(Figure 1)** will be included in the study.

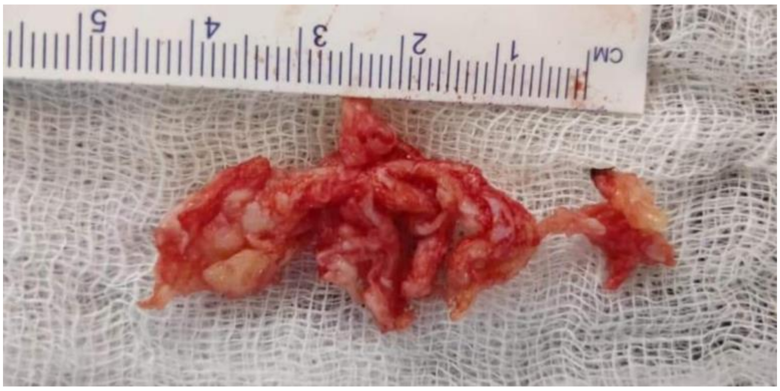

Figure 1 – Disc Material Obtained After Lumbar Discectomy.

Patients with a previous history of intervertebral disc infection and/or with signs of spondylodiscitis, patients previously submitted to procedures in the intervertebral disc collection area through discectomy, will not be included in the study. Also, patients using oral or parenteral antibiotics in the period of fifteen days prior to the date of the surgical procedure in which the samples will be collected will not be included.

CLINICAL CHARACTERIZATION

Demographic data will be collected from all participants, as well as an inventory of their comorbidities and past pathological history.

For the purposes of selecting participants in this project, chronic low back pain will be considered all that lasts longer than three months, regardless of association with irradiation to the lower limbs and/or neurological signs resulting from spinal or radicular compression.

For the characterization of spondylodiscitis, 1) fever will be considered, 2) asthenia or 3) weight loss, 4) presence of laboratory alterations suggestive of active infection (leukocytosis or bastonemia) or 5) magnetic resonance imaging (MRI) with suggestive signs of infection (Figure 2).

Participants will be graded according to the Visual Analogue Pain Scale (VAS) and according to the Oswestry functional score (ATTACHMENT 1). (34)

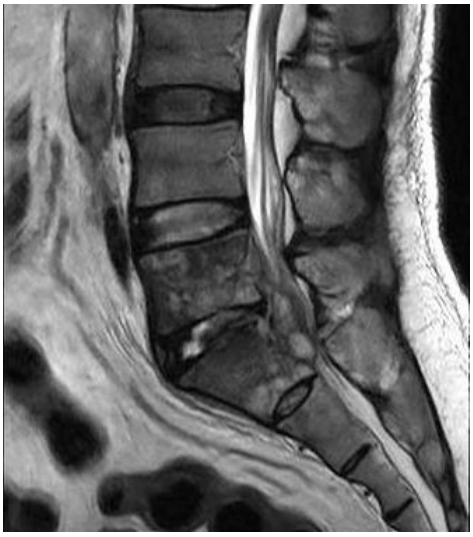

Figure 2 – NMR of the Lumbar Spine in T2 with Spondylodiscitis of the L5-S1 segment.

IMAGING CHARACTERIZATION

The presence of Modic alterations in the MRI exams will be documented for each participant, as well as these same images of the approached discs will be graded according to the Pfirrmann classification (35, 36).

ANTIBIOTIC PROPHYLAXIS AND THE TIME OF SAMPLE COLLECTION

Intravenous antibiotic therapy will be administered during anesthetic induction for the surgical procedure in which samples will be collected. The antimicrobial used as a protocol in the hospitals where the surgeries will be performed is cefazolin, at a dose of 1g, repeated every 4 hours for the duration of the surgery. The antibiotic prophylaxis regimen will end with skin closure and will not be extended for an additional time after surgery.

CLINICAL SPECIMEN COLLECTED

As soon as the patients are admitted to the operating room, during anesthetic induction and before the intravenous infusion of surgical antibiotic prophylaxis, a 10ml

aliquot of blood will be collected (Figure 3) by venoclysis of the upper limb, which will be reserved in a tube containing clot for further serum separation.

Despite not being very specific due to the influence they may suffer from other infections and non-infectious inflammatory diseases, including extra-articular infections, in addition to the laboratory method used, these markers are excellent predictors of the absence of infection, when both are negative. (37)

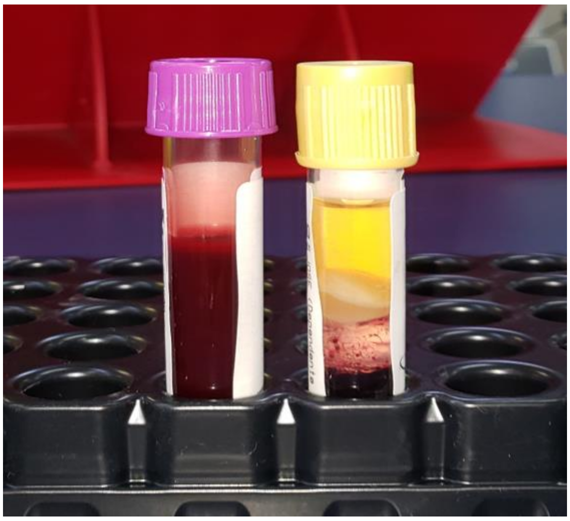

Figure 3 - Skin Swab Collection from the Surgical Site

Immediately before skin asepsis, a skin swab will be collected from the area where the surgical incision will be made. After performing asepsis with 2% chlorhexidine gluconate degerming solution and 0.5% chlorhexidine gluconate alcoholic solution, a new swab will be collected from the same region before the incision is made (Figure 4).

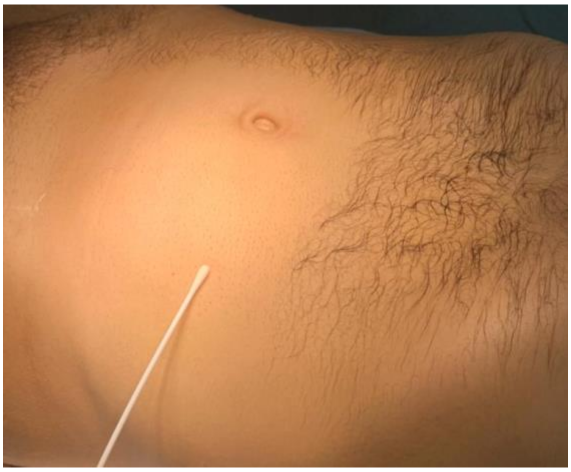

Figure 4 - Skin Swab Collection from the Surgical Site

Finally, during the surgery, 5 intervertebral disc fragments will be collected in each approached segment (Figure 1). In the same act, fragments of muscle-ligament tissue (Figure 5) surrounding the intervertebral disc will also be collected (two fragments per collected disc level).

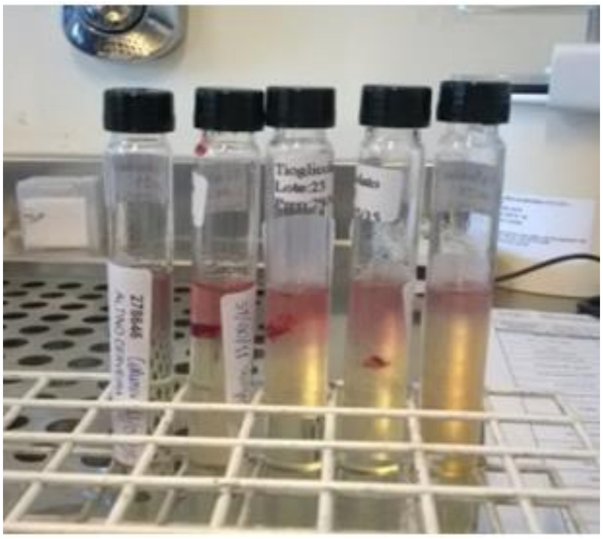

Figure 5 – Musculoligamentous Tissue Fragments Collected

After laboratory analyses, the collected clinical specimen samples will finally be discarded.

PROCESSING OF SAMPLES

SKIN SWAB

After collecting the skin swab, it will be placed in Stuart medium and transported to the Laboratory of Anaerobic Biology (UFRJ), being opened only at the time of sowing on blood agar plates (5% defibrinated sheep blood, base of blood agar – 40 g/L, agar – 5 g/L) and anaerobic blood agar (5% defibrinated sheep blood, blood agar base – 40 g/L, agar – 5 g/L, hemin – 10 mL / L and menadione – 5 drops/L). After sowing in solid media, the swab will be inoculated in thioglycolate medium where it will remain for 14 days. One of the plates will be incubated in a capnophilic atmosphere (5% to 10% carbon dioxide), while the other will be incubated in strict anaerobiosis using an anaerobic jar or Glove Box – Coy Labs USA (atmosphere containing 10% carbon dioxide, 10% hydrogen and 80% nitrogen). Plates will be read after 24 hours of incubation and, in case of growth, colonies will be identified. In the absence of growth, they will be reincubated and a new reading performed after 72 hours. Vials of thioglycolate containing skin swabs will be kept in a bacteriological oven (35°C to 37°C) for 14 days.

MUSCLE-LIGAMENTARY TISSUE AND INTERVERTEBRAL DISC

Clinical specimens of musculoskeletal tissue will be collected by the same team of spine surgeons (orthopedists and neurosurgeons) who work in the three hospitals where the surgeries will be performed. A set of sterile Kelly tweezers will be used exclusively for collection, one for each tissue fragment collected. A total of 2

specimens of surrounding musculo-ligamentous tissue and 5 specimens of intervertebral disc will be obtained for each level of discectomy.

Immediately after collection, the Kelly forceps with tissue fragment will be delivered to a member of the research team present in the operating room. Each specimen will be inoculated in a flask containing thioglycolate culture medium (MERCK, Brazil), (casein peptone – 19.5 g/L, yeast extract – 5.0 g/L, glucose – 6.0g/L, chloride sodium – 2.7 g/L, L-cystine – 0.5 g/L, sodium thioglycolate – 0.5 g/L, agar – 0.75 g/L) and beads and glass.

The vials will be identified with the initials of the study participant and the type and clinical specimen. After collection, the vials containing the clinical specimens will be kept at room temperature and transported in a special bag to the laboratory within a maximum period of 2 hours.

In the laboratory, each tube containing the clinical specimen inoculated in thioglycolate medium will be subjected to vortexing (Even EVX2800-BI, Brazil) for 15 seconds (Figure 6).

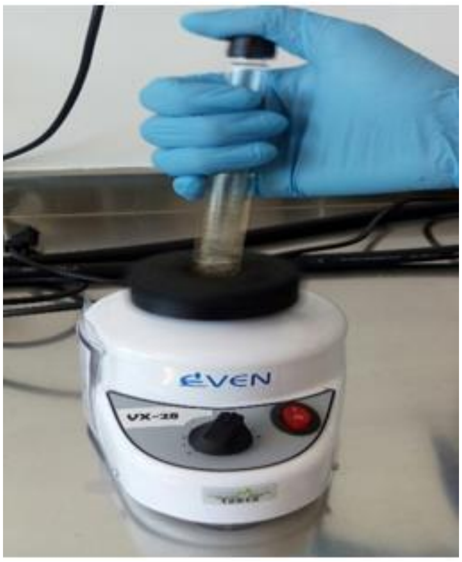

Figure 6 - Vortex of Clinical Specimens and Glass Beads

All tubes will be kept in a bacteriological oven (35°C to 37°C) and after 24h, the first subculture will be performed. With the aid of a bacteriological loop of 100 microliters (µL), an aliquot of thioglycolate will be removed and seeded in blood agar (5% defibrinated sheep blood, blood agar base – 40 g/L, agar – 5 g/L) and anaerobic blood agar (5% defibrinated sheep blood, blood agar base – 40 g/L, agar – 5 g/L, hemin – 10 mL/L and menadione – 5 drops/L). The first plate will be incubated in a capnophilic atmosphere (5% to 10% carbon dioxide) and the second in strict anaerobiosis using an anaerobic jar or Glove Box – Coy Labs USA (atmosphere containing 10% carbon dioxide, 10% hydrogen and 80% nitrogen). The reading of the plates will be performed after 24 hours and in case of growth, the colonies will be identified.

In the absence of growth, they will be reincubated and a new reading performed after 72 hours. The same procedure will be used for all vials of thioglycolate (totaling 5 vials). The thioglycolate tubes containing the clinical specimens will be kept in an

oven for 14 days. Subcultures will be performed after 72 hours and always every 3 days, with the last *subculture* performed on the 14th day of incubation in the same solid culture media and atmospheres used in the first 24 hours.

BACTERIOLOGICAL IDENTIFICATION

Species identification will be performed by mass spectrometry with Bruker Biotyper® MALDI-TOF equipment, Germany [Matrix-assisted laser desorption/ionization time-of-flight mass spectrometry] located at Instituto de Microbiologia Paulo de Goes. The score used for a reliable identification is 2,000 and 3,000 for the MALDI-TOF equipment, according to the manufacturer's instructions. The interpretation of the score is done as follows: for scores in the range of 2,300 to 3,000, the identification of the genus and species is highly secure, from 2,000 to 2,299, the identification of the genus is secure and the identification of the species probable. Genus identification is likely with a score of 1,700 to 1,999, and from 0,000 to 1,699, the microorganism was not identified.

GENOTYPIC CHARACTERIZATION

The microorganisms isolated from cultures will be subjected to PCR analysis performed in two phases, as described by Bernard et al. (28) The first to confirm the presence of bacteria (target and non-target) by using species-specific primers targeting the rRNA-16s and a second one, Multiplex_PCR, targeting the virulence and oxidative gens.

WHOLE GENOME SEQUENCING

For the WGS, the DNA of the isolated C. acnes strains will be obtained with the Qiagen Blood & Tissue DNA extraction kit (Qiagen®, USA) and subsequent sample purification using RNase. The extracted and purified DNA will be dosed, and adjusted to a concentration of 0.2 ng/µL. The ends of its two ribbons will be fragmented and tagged (forward and reverse 5'--3'). This will form fragments of different sizes. This data will be used to create a database on the Nextera Flex Kit platform (Ilumina®, USA). The samples will then be multiplexed into a cell stream and run on the NextSeq sequencer (Ilumina®, USA) using paired sequencing to generate files in fastq format files, which will then be filtered using the Trimmomatic tool version 0.36, as recommended by Bolger et al.(54) This step will be critical for controlling the quality of the analysis because it reduces the possibility of errors during sequencing and allows the use of more reliable reading codes. The obtained fragments will be sequenced, and their quality will be evaluated with the FastQC tool version 0.11.9. The RedDog software version 1.11 will be used to identify single nucleotide polymorphisms (SNPs) using the GCA_000008345.1 C. acnes reference strain deposited in the GenBank NCBI [https://www.ncbi.nlm.nih.gov/nuccore/] as a comparison. Finally, the PATRIC software version 3.5.21 will be used to create a complete genome phylogeny from the concatenation of SNPs.

PROTEOMICS

The sodium dodecyl sulfate polyacrylamide gel electrophoresis (SDS-PAGE) technique and (2) in a solution technique will be used for proteomic analysis in this study. Thioglycolate culture mediums will be centrifuged, and the sediment obtained will be washed in phosphate buffer and centrifuged again. The sediment will be dissolved in a lysis solution containing 8 M urea, 2% 3-[(3-cholamidopropyl) dimethylammonio]-1-propanesulfonate, and 40 mM Tris (hydroxymethyl)

aminomethane. This lysis will be complemented with the use of glass beads under a Bead Beater agitation (Biospec Product®, USA) (3 × 20 s with intervals of 20 s) followed by cooling for 5 min on an ice bath, as described by Shah et al.(55) Protein concentration will be measured and 20 µg will be used for both the gel and solution techniques. The SDS-PAGE gel-based technique will be used to evaluate the profile of the proteins present in the extract. Proteins will be removed from the gel with a sterile scalpel,

QUALITY CONTROL OF INPUTS

To control the sterility of the thioglycolate broth, sterile 0.9% saline solution will be used and to evaluate the recovery capacity of the facultative and strict anaerobic microorganisms, the strains P. aeruginosa ATCC 27853 and B. fragilis 25285 will be used. always carried out with each new batch of prepared culture medium.

Quality control of the solid media used in the study; Agar with 5% defibrinated sheep blood and anaerobic blood agar will also be performed to evaluate the recovery capacity of facultative microorganisms and strict anaerobes with strains P. aeruginosa ATCC 27853 and B. fragilis 25285, respectively and always with each new batch.

QUESTIONNAIRES

Questionnaires on pain (VNS), functionality (ODI), and quality of life (EQ-5D) will be given at recruitment as well as at 1, 3, and 6 months postoperatively. A professional who is not involved in the study will collect all questionaires. Follow-up clinical visits will be carried out at 1, 3, and 6 months postoperatively, with acceptance deviation of 7, 14, and 21 days, respectively.

ETHICAL ASPECTS

The project will be carried out in accordance with the ethical standards of the Ethics Committee and Research with Human Beings (CEP) of the HUGG. All patients whose biological materials will be analyzed will receive clarification on the objectives of the research proposed here and will sign the Informed Consent Form (TCLE), agreeing with the availability of samples for further laboratory analysis (ANNEX 2). Collections will only start after due approval from the Institution's CEP.

EXPECTED RESULTS

With the collection and isolation techniques employed, the authors hope to identify a higher prevalence of colonization of the intervertebral discs by C. acnes than those presented by other authors until the moment of proposing this project.

RESEARCH RISKS

The risks of the research for the participants are those related to the surgical act where the samples will be collected, among which are mentioned: changes in skin sensitivity or mobility restrictions, numbness in the legs, changes in urination control, local pain, swelling , edema, heat, redness, surgical wound infection and non-consolidation.

The information obtained through the research will be confidential and the researchers will ensure the confidentiality of the patients' participation. The collection of clinical specimens will be numbered and the name of the participants will not appear on any container to be stored.

FINANTIAL SUPPORT

The project will be financed by the responsible researcher, who will pay for the transportation of the samples. The culture media and reagents used in the laboratory analyses, as well as the bench fee for the microbiology technician will be funded by the Laboratory of Anaerobic Biology of the Instituto de Microbiologia Paulo de Góes through funding received by funding agencies CAPES, CNPq and FAPERJ. The estimated values are listed in the Research Budget topic, part of this document.

REFERÊNCIAS

1. Mendonça AG, Oliveira VC, Fonseca LS, Oliveira MX. Custos diretos da dor lombar em hospitais financiados pelo Sistema Único de Saúde. Revista Pesquisa em Fisioterapia. 2021;11(1).
2. Nascimento PR, Costa LO. Low back pain prevalence in Brazil: a systematic review. Cad Saude Publica. 2015;31(6):1141-56.
3. Vasconcelos FH, Araújo GCd. Prevalence of chronic pain in Brazil: a descriptive study. Brazilian Journal Of Pain. 2018;1(2).
4. Katz JN. Lumbar disc disorders and low-back pain: socioeconomic factors and consequences. J Bone Joint Surg Am. 2006;88 Suppl 2:21-4.
5. Taher F, Essig D, Lebl DR, Hughes AP, Sama AA, Cammisa FP, et al. Lumbar degenerative disc disease: current and future concepts of diagnosis and management. Adv Orthop. 2012;2012:970752.
6. Parenteau CS, Lau EC, Campbell IC, Courtney A. Prevalence of spine degeneration diagnosis by type, age, gender, and obesity using Medicare data. Sci Rep. 2021;11(1):5389.
7. Gorth DJ, Shapiro IM, Risbud MV. Discovery of the drivers of inflammation induced chronic low back pain: from bacteria to diabetes. Discov Med. 2015;20(110):177-84.
8. Choi YS. Pathophysiology of degenerative disc disease. Asian Spine J. 2009;3(1):39-44.
9. Qiu C, Wu X, Bian J, Ma X, Zhang G, Guo Z, et al. Differential proteomic analysis of fetal and geriatric lumbar nucleus pulposus: immunoinflammation and age-related intervertebral disc degeneration. BMC Musculoskelet Disord. 2020;21(1):339.
10. Abdollah V, Parent EC, Su A, Wachowicz K, Battie MC. The effects of axial loading on the morphometric and T2 characteristics of lumbar discs in relation to disc degeneration. Clin Biomech (Bristol, Avon). 2021;83:105291.
11. Oichi T, Taniguchi Y, Oshima Y, Tanaka S, Saito T. Pathomechanism of intervertebral disc degeneration. JOR Spine. 2020;3(1):e1076.
12. Weiner SS, Nordin M. Prevention and management of chronic back pain. Best Pract Res Clin Rheumatol. 2010;24(2):267-79.
13. Manniche C, O'Neill S. New insights link low-virulent disc infections to the etiology of severe disc degeneration and Modic changes. Future Sci OA. 2019;5(5):FSO389.
14. Jiao Y, Lin Y, Zheng Y, Yuan Y, Chen Z, Cao P. The bacteria-positive proportion in the disc tissue samples from surgery: a systematic review and meta-analysis. Eur Spine J. 2019;28(12):2941-50.

15. Yuan Y, Chen Y, Zhou Z, Jiao Y, Li C, Zheng Y, et al. Association between chronic inflammation and latent infection of Propionibacterium acnes in non-pyogenic degenerated intervertebral discs: a pilot study. Eur Spine J. 2018;27(10):2506-17.
16. Chen Y, Wang X, Zhang X, Ren H, Huang B, Chen J, et al. Low virulence bacterial infections in cervical intervertebral discs: a prospective case series. Eur Spine J. 2018;27(10):2496-505.
17. Stirling A, Worthington T, Rafiq M, Lambert PA, Elliott TSJ. Association between sciatica and Propionibacterium acnes. The Lancet. 2001;357(9273):2024-5.
18. Ohtori S, Koshi T, Yamashita M, Yamauchi K, Inoue G, Suzuki M, et al. Existence of pyogenic spondylitis in Modic type 1 change without other signs of infection: 2-year follow-up. Eur Spine J. 2010;19(7):1200-5.
19. Arndt J, Charles YP, Koebel C, Bogorin I, Steib JP. Bacteriology of degenerated lumbar intervertebral disks. J Spinal Disord Tech. 2012;25(7):E211-6.
20. Ganko R, Rao PJ, Phan K, Mobbs RJ. Can bacterial infection by low virulent organisms be a plausible cause for symptomatic disc degeneration? A systematic review. Spine (Phila Pa 1976). 2015;40(10):E587-92.
21. Urquhart DM, Zheng Y, Cheng AC, Rosenfeld JV, Chan P, Liew S, et al. Could low grade bacterial infection contribute to low back pain? A systematic review. BMC Med. 2015;13:13.
22. Astur N, Martins DE, Wajchenberg M, Ferretti M, Menezes FG, Doi AM, et al. Subclinical Propionibacterium acnes infection estimation in the intervertebral disc (SPInE-ID): protocol for a prospective cohort. BMJ Open. 2017;7(11):e017930.
23. Valanne S, McDowell A, Ramage G, Tunney MM, Einarsson GG, O'Hagan S, et al. CAMP factor homologues in Propionibacterium acnes: a new protein family differentially expressed by types I and II. Microbiology (Reading). 2005;151(Pt 5):1369-79.
24. McDowell A, Valanne S, Ramage G, Tunney MM, Glenn JV, McLorinan GC, et al. Propionibacterium acnes types I and II represent phylogenetically distinct groups. J Clin Microbiol. 2005;43(1):326-34.
25. Mollerup S, Friis-Nielsen J, Vinner L, Hansen TA, Richter SR, Fridholm H, et al. Propionibacterium acnes: Disease-Causing Agent or Common Contaminant? Detection in Diverse Patient Samples by Next-Generation Sequencing. J Clin Microbiol. 2016;54(4):980-7.
26. Aubin GG, Portillo ME, Trampuz A, Corvec S. Propionibacterium acnes, an emerging pathogen: from acne to implant-infections, from phylotype to resistance. Med Mal Infect. 2014;44(6):241-50.
27. Yuan Y, Zhou Z, Jiao Y, Li C, Zheng Y, Lin Y, et al. Histological Identification of Propionibacterium acnes in Nonpyogenic Degenerated Intervertebral Discs. Biomed Res Int. 2017;2017:6192935.
28. Butler-Wu SM, Burns EM, Pottinger PS, Magaret AS, Rakeman JL, Matsen FA, 3rd, et al. Optimization of periprosthetic culture for diagnosis of Propionibacterium acnes prosthetic joint infection. J Clin Microbiol. 2011;49(7):2490-5.
29. Minassian AM, Newnham R, Kalimeris E, Bejon P, Atkins BL, Bowler IC. Use of an automated blood culture system (BD BACTEC) for diagnosis of prosthetic joint infections: easy and fast. BMC Infect Dis. 2014;14:233.
30. Shannon SK, Mandrekar J, Gustafson DR, Rucinski SL, Dailey AL, Segner RE, et al. Anaerobic thioglycolate broth culture for recovery of Propionibacterium acnes from shoulder tissue and fluid specimens. J Clin Microbiol. 2013;51(2):731-2.
31. Tande AJ, Patel R. Prosthetic joint infection. Clin Microbiol Rev. 2014;27(2):302-45.
32. Capoor MN, Lochman J, McDowell A, Schmitz JE, Solansky M, Zapletalova M, et al. Intervertebral disc penetration by antibiotics used prophylactically in spinal surgery:

implications for the current standards and treatment of disc infections. Eur Spine J. 2019;28(4):783-91.
33. Instituto Brasileiro de Geografia e Estatística. Censo Demográfico 2010, [acesso em 27 mar 2021]. Disponível em: https://cidades.ibge.gov.br/brasil/rj/rio-de-janeiro/panorama.
34. Vigatto R, Alexandre NM, Correa Filho HR. Development of a Brazilian Portuguese version of the Oswestry Disability Index: cross-cultural adaptation, reliability, and validity. Spine (Phila Pa 1976). 2007;32(4):481-6.
35. Zhang YH, Zhao CQ, Jiang LS, Chen XD, Dai LY. Modic changes: a systematic review of the literature. Eur Spine J. 2008;17(10):1289-99.
36. Urrutia J, Besa P, Campos M, Cikutovic P, Cabezon M, Molina M, et al. The Pfirrmann classification of lumbar intervertebral disc degeneration: an independent inter- and intra-observer agreement assessment. Eur Spine J. 2016;25(9):2728-33.
37. Enayatollahi MA, Parvizi J. Diagnosis of infected total hip arthroplasty. Hip Int. 2015;25(4):294-300.

TIMELINE

RESEARCH PROJECT: COLONIZATION OF INTERVERTEBRAL DISCS BY CUTIBACTERIUM ACNES IN PATIENTS WITH CHRONIC LUMBAR PAIN

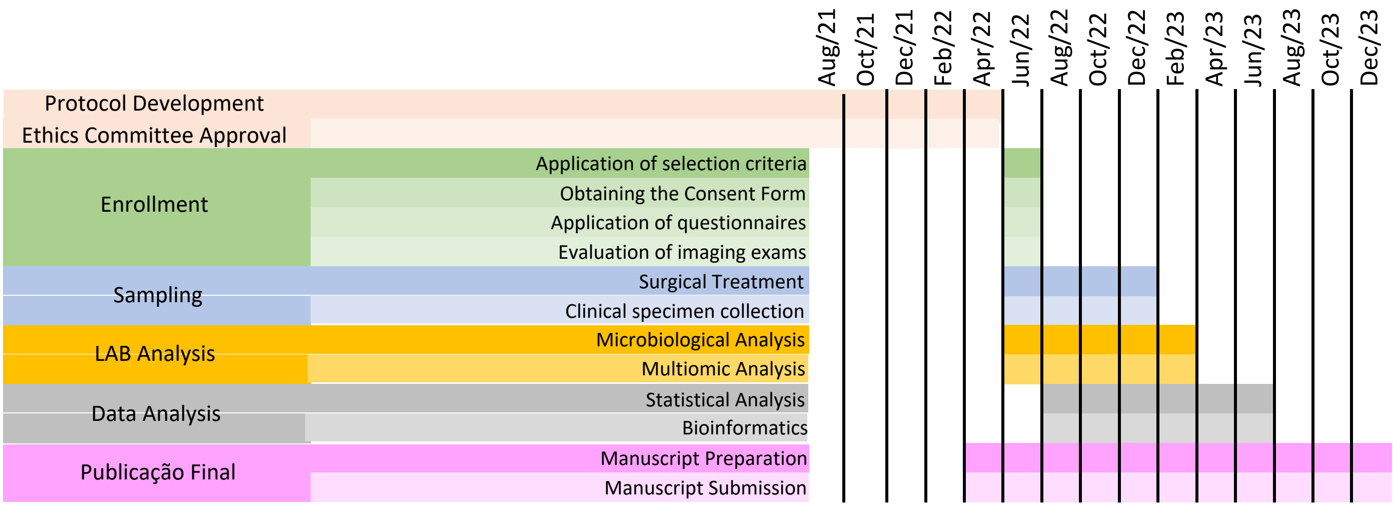

CONSENT FORM

(According to the Norms of the Resolution of the National Health Brazilian Council nº 466 of 12/12/2012)

You are being invited to participate in the research "**COLONIZATION OF INTERVERTEBRAL DISCS BY *Cutibacterium acnes* IN PATIENTS WITH CHRONIC LUMBAR PAIN**" which will be carried out at Hospital São Matheus, in partnership with the Gaffrée e Guinle University Hospital and the Health Sciences Center of the Federal University of Rio de Janeiro (UFRJ).

You were selected based on the characteristics of your illness at the hospital where you are being monitored. Your participation is not mandatory, so you can withdraw your consent at any time. Your refusal will not harm your relationship with your attending physician, with the researchers or with the institutions involved in the research.

The purpose of this study is to analyze tissue fragments that will be removed from your spine during your spine surgery. These fragments are obligatorily removed during the procedure to which you will be submitted to be discarded, so that their collection will not incur costs, modifications in the surgical technique or increase in the duration of the surgery. The analysis of these fragments will allow us to identify the presence of bacteria in these tissues, especially *Cutibacterium acnes*. After laboratory analysis of the tissue fragments, they will finally be discarded.

The benefits related to your participation will mainly be a better understanding of the process of intervertebral disc degeneration and infections related to the surgical procedure. There are no discomforts or additional risks to your health arising from your participation in this research, in addition to those related to the surgical act to which you will be submitted, which are: changes in skin sensitivity or mobility restrictions, numbness in the legs, changes in micturition control, local pain, swelling, edema, heat, flushing, in short, normal alterations to surgical trauma, which will completely or partially regress with time, with no specific deadline for this to occur. It is important that you know that all spinal surgery has around a 2% (two percent) chance of infection and a 4% (four percent) chance of non-consolidation, which may require new interventions to perform dressings , removal of metallic implants or revisions thereof.

The information obtained through this research will be confidential and we assure confidentiality about your participation. The collections will be numbered and your name will not appear on any container to be stored, but it is important that you also know that your clinical data collected through questionnaires during the research may be breached in the event of any event threatening your health.

This Term was drawn up in three copies, one for you (participant), another for the researcher and a third that will be attached to your medical record. This term contains the telephone number and address of the main researcher and the Research Ethics Committee – CEP where this research will be registered. You will be able to clarify your doubts about the project and your participation, now or at any time through the phone numbers or email address available in this Term. You may also withdraw from participating at any time and withdraw your Consent Form, without prejudice to your treatment.

Rio de Janeiro,______de___________________de __________

Main researcher: Professor Vinícius Magno da Rocha (DECIGE) - Register 1847765
Address: R. Mariz e Barros, 775 - Maracanã, Rio de Janeiro - RJ. Orthopedics Service - First Floor
E-mail: professorviniciusmagno@edu.unirio.br

In case of doubt regarding the ethical conduct of the study, contact the Research Ethics Committee of the Gaffrée e Guinle University Hospital.
Address: Rua Mariz e Barros, Nº 775, 4º andar (fundos), Maracanã, Rio de Janeiro/RJ
E-mail: cephugg@gmail.com
Telefone: (21) 2264-5177

I,____, born in _____________ and bearer of nº_____________________________, after reading (or listening to the reading) of this document and having had the opportunity to talk to a member of the research team to clarify all my doubts, I believe I am sufficiently informed, making it clear to me that my participation is voluntary and that I can withdraw this consent at any time without penalties or loss of any benefit. I am also aware of the objectives of the research, the procedures to which I will be submitted, the possible damages or risks arising from them and the guarantee of confidentiality and clarifications whenever I wish. In view of the above, I express my voluntary agreement to participate in this study.

____
Signature of the volunteer or his legal representative

____
Signature of the person responsible for obtaining the Consent Form
